# Exploring metric dimension of nanosheets, nanotubes, and nanotori of SiO_2_

**DOI:** 10.1371/journal.pone.0316376

**Published:** 2025-03-04

**Authors:** Umar Farooq, Wasim Abbas, Faryal Chaudhry, Muhammad Azeem, Bandar Almohsen

**Affiliations:** 1 Department of Mathematics and Statistics, The University of Lahore, Lahore, Pakistan; 2 Department of Mathematics, Riphah International University, Lahore, Pakistan; 3 Department of Solids and Structures, School of Engineering, The University of Manchester, Manchester, United Kingdom; 4 Department of Mathematics, College of Science, King Saud University, Riyadh, Saudi Arabia; International Iberian Nanotechnology Laboratory, PORTUGAL

## Abstract

This work investigates the metric dimension (MD) and edge metric dimension (EMD) of SiO_2_ nanostructures, specifically nanosheets, nanotubes, and nanotorii. The metric dimension describes the minimum number of vertices required to uniquely identify every other point in a graph. In contrast, the edge metric dimension is the minimum number of vertices needed to distinguish each edge. Understanding these dimensions is essential for characterizing the geometric and structural properties of nanoparticles. Using graph theory techniques, we compute the MD and EMD of various SiO_2_ nanostructures to elucidate their unique geometries and configurations. Our findings offer precise formulas for these dimensions, critical for designing and optimizing SiO_2_-based materials with targeted properties. This study provides valuable insights for applications in chemistry, materials science, and nanotechnology, where knowledge of structural characteristics at the nanoscale is crucial.

## Introduction

Chemical graph theory is a branch in graph theory with the mathematics and its specialty. Thus, the main objective of chemical graph theory is to look at various structures of chemicals by representing it graphically. The use of direct analysis might be restricted especially when the chemical structures are large and complex in nature. This is made easier by chemical graph theory, since the just described structures are accounted for as molecular graphs having the atoms as the vertices and the bonds between the atoms as the edges.

This framework employs a mathematical model that tends to facilitate the analysis of the physical properties of the chemical structure by determining every atom out of it to be a vertex. For identifying the complete vertex set it is required to choose a subset of atoms (vertices) and for each of them a definite position regarding chosen vertices is defined [1]. This concept is known as the metric basis for the graph theory and as resolving set (metric generator) in the applied graph theory [2]. Hernando et al. [3] proposed fault tolerance in metric generators to decrease the chance of system failure should a part of the generator fail.

Furthermore, bonds are not limited to uniquely distinguishing atomic locations; they can also be utilized to characterize the structure. To this end, Kelenc et al. introduced the EMD, a variant of the MD that focuses on uniquely identifying the graph’s edges in non-trivial connected graphs [4]. Similar to resolving sets, Liu et al. introduced the idea of fault tolerance in edge resolving sets [5].

The practical applications of MD have been beneficial to many fields, such as robotic navigation [6], geographic routing protocols [7], connected joints in networks and chemistry [8], telecommunications [9], combinatorial optimization [10], and network discovery and verification [9]. The NP-hardness and computational complexity of resolvability parameters are investigated in a number of works [11,12].

The MD has been extensively studied for a variety of chemical structures because to its numerous applications in the chemical sciences. Reference [13] discusses the vertex resolvability of H-Naphthalenic nanotubes and $VC_{5}C_{7}.$ [14] contains the least resolving sets of silicates star networks, and [15] provides upper bounds on the fewest resolving sets of cellulose networks. The MD of the 2*D* lattice of boron nanotubes (Alpha) is examined in [16]. Additionally, a number of different MD variants, such the EMD, FTMD, and FTEMD (fault-tolerant edge metric dimension), have been investigated for a range of graph families and chemical structures.

A great deal of chemistry is based on graph theory, including quantitative structure-activity relationships, biological activity forecasting, topological analysis of chemical compounds, quantum chemistry, isomer counting, spectroscopy, graph polynomials for structural analysis, nuclear spin statistics, NMR spectroscopy, and toxicity prediction techniques. The study of structure-property interactions in mesoporous materials, fullerenes, and nanomaterials [17–33] might also benefit from its use.

Graph theory has numerous applications in chemistry, such as in the characterization of chemical structures, prediction of biological activities, and the study of nanomaterials. Quantitative structure-activity and property relationships (QSAR/QSPR) utilize the molecular connectivity of chemical compounds and their properties, forming the basis for computer-aided drug discovery and predictive toxicology. Successful applications of QSAR/QSPR have led to the development of various topological descriptors for molecules, periodic structures, fullerenes, lattices, proteomes, and nanomaterials.

Fundamental mathematical methods such as graph reductions, iterative techniques, recursive procedures, and tree-pruning algorithms have been employed to derive a range of topological properties. These characteristics include distance polynomials, matching polynomials, and spectral polynomials of complex lattices, fullerenes, organic polymers, and nanotubes [34,35]. The degree and distance parameters significantly influence intermolecular interactions, which in turn affect various physicochemical properties of compounds. These properties include boiling points, melting points, vapor pressures, dermal penetration, octanol partition coefficients, chromatographic retention indices, and 2D-gel electrophoresis patterns of proteomes [36].

The computational ease of topological approaches makes them commonly employed, even if more sophisticated quantum chemical and bio-descriptors and quantum molecular dynamics simulations are required for accurate predictions of chemical and biological properties.

SiO2, or silicon dioxide, is an essential substance used in many fields, including biology and the semiconductor industry. In medications, it serves as an inactive filler, absorbent, and anti-caking agent. The customizable particle size and specific surface area of silica-based nanomaterials, their abundance of Si-OH bonds for functionalization [52], their chemical and thermal stability, their high drug loading capacity, and their sustained drug release—all of which increase drug bioavailability—have all attracted attention in recent years [37–41].

## Importance of metric dimension in various fields

The MD is indeed important in graph theory and proves to be very helpful in understanding the features and functionality of various systems represented graphically. More precisely, it indicates the smallest number of vertices by which it is possible to describe all the other vertices in that graph. This idea is used in many fields and areas in engineering and computing, artificial intelligence, root management, town planning, and building development[42,43].

### Engineering applications

In the field of engineering, this concept of MD plays a key role, especially in the design and the optimization of numerous networks including the sensor networks, the communication systems, as well as the transportation infrastructures. For instance, in sensor network identification, the MD enables one to know the best place to fix sensors to get good coverage without having to fix more sensors than needed in the same region. Being accurate also contributes to the savings of cash as well as improving the rate of tracking and gathering data. Likewise, in transportation networks, the awareness of the MD enhances the route and schedule assignment since all the points on the network can be uniquely distinguished and connected, which enhances the overall performance of the logistics and transportation systems [44,45].

### Applications in artificial intelligence and machine learning

Within the fields of machine learning and artificial intelligence, the MD is an important concept that improves the efficiency of such algorithms taken place on the graph. As many AI applications are based on the social network analysis, pattern recognition, or recommendation systems, the graph concepts are widely used to describe relations between nodes. In particular, based on the MD, it is possible to enhance the performance of algorithms that need the identification of nodes or edges within a graph. For instance, in social network analysis, the MD can assist in defining a priority target – a single person or a group of persons – thus, the information or the marketing strategy could be targeted directly to the [46,47].

### Relevance in computing and network design

The MD is also used in computing, especially in the development of communication networks and structures; and in the analysis of algorithms. In the process of designing a network, there is such a concept as the MD of networks which is important while creating routing protocols and defining means of error detection. For example, in distributed computing environments each node has to be uniquely identifiable and for that purpose, MD is used which is a crucial factor in realizing the concept of fault tolerance and accidental data transfer. Moreover, in the efficiency of constructing data structures, the MD can enhance search strategies, and decrease the time and computational cost for identifying certain objects in great databases.

### Application in root management systems

In RMS especially in agricultural and environmental sciences, there is much benefit of the MD where it brings out structure stability of root system and distribution of the resources. Applying the ideas of the MD to root systems represented as the graphs allows the researchers to investigate the resource distribution and changes in the plants depending on the conditions. It is essential for maximizing yield, enhancing the quality of soil, and enhancing mechanisms for sustainable use of the soil resources in crop production. Furthermore, the knowledge of the MD of root networks plays an important role in the development of stronger and more considerable images of ecosystems that can be more protected from external conditions such as drought or erodibility of the soil [50,51] .

### Impact on town planning and urban development

In town planning the MD plays a critical role in the determination of efficient mechanisms of the urban structures that are hardy. As applied to transport, utility, and communication networks, problems of planning can be solved using graph theory; in doing so, the MD can be used to guarantee unique identification and accessibility of critical nodes of intersection or service points. This helps in the development of urban structures that are complex and integrated with networks that are easy to follow and can accommodate the newfound population. Moreover, the MD helps in preparing the strategies for the management of disasters and it is used to determine which components of infrastructures need to be safeguarded or even reinforced [52,53].

### Application in building development and architecture

Regarding the MD in building development and architecture, it refers to the design of buildings and their ways of construction in the most efficient manner possible. In this case, the MD will help the architect or engineers who have graph modeled a building to identify the unique parts of the structure’s elements such as the beams, columns, and load-bearing walls, and place them most efficiently and effectively. This results in the improvement of safety, stability, and functionality of the building in question. Also, the MD can be used in building smart solutions where environmental indicators, air and energy controls, surveillance, and other related aspects are implemented in buildings[54].

The work with the given notion of the MD became possible after leaving the abstraction sphere, which allowed for applying it to various fields and receiving significant advantages. By providing a quantitative way to identify elements within the systems the MD helps to solve problems of organization and optimization in engineering, artificial intelligence, computing, root management, town planning, and building development. Thus the MD knowledge and theories will be essential as systems in these fields advance into higher orders to boost innovation and the structure of essential infrastructures and technologies [55].

in this paper, we look at the MD of Nanosheets, Nanotubes, and Nanotori of $SiO_{2}$

### Previous work on SiO_2_ nanosheets, nanotubes, and nanotori

Silicon dioxide (SiO_2_) nanostructures, including nanosheets, nanotubes, and nanotorii, have been extensively studied due to their unique structural and functional properties. SiO_2_ nanosheets, for instance, have garnered attention for their high surface area, stability, and reactivity, which make them ideal for applications in catalysis, drug delivery, and electronic devices [37,38]. The layered structure of SiO_2_ nanosheets also facilitates the attachment of functional groups, which has further expanded their applications in biochemical and electronic fields [39,40]. Nanotubes of SiO_2_, on the other hand, offer a hollow core that has proven beneficial in areas such as drug delivery and nanofluidics, where controlled release and selective adsorption are required [41,42]. SiO_2_ nanotori, though less commonly studied, have shown promise for applications in optical materials and photothermal drug delivery, benefiting from their unique toroidal structure and potential for plasmonic resonance [43,44].

Recent research has focused on understanding the topological and geometrical properties of these SiO_2_ nanostructures to optimize their functionality. For example, studies on the electronic properties of SiO_2_ nanosheets and nanotubes have shown that their structure affects band gap and charge transport properties, which are crucial for applications in sensors and transistors [45,46]. In the same way, the mechanical properties of SiO_2_ nanotori have been determined to check their resilience and suitability for applications in stressed environments [47]. Topological indices and graph-theoretic methods have been previously applied to check the structural symmetry of these nanostructures, but studies did not address their metric dimension comprehensively.

### Metric dimension and edge metric dimension in SiO_2_ nanostructures

This research has not been conducted previously as it investigates for the first time the *metric dimension (MD)* and *edge metric dimension (EMD)* of SiO_2_ nanosheets, nanotubes and nanotorii. The MD is the smallest set of vertices such that each vertex of the graph apart from this set can be uniquely identified knowing the location of the vertices in this set. In the case of the EMD, edges are the attributes of the vertices and it can be extended to edges like vertex identification. In this way, the SiO_2_ structures listed above are characterized topologically by the concepts of MD and EMD enabling the determination of the resolution and their structural arrangement. Previously only topological indices were computed, which were rather ‘primitive’ in the academic language of indices. In contrast, this work provides MD and EMD – which are essential for the robust characterization of SiO_2_ nanostructures.

A profound difference between our work from previous studies seems to be the focus on metric dimensions - the aspect that enables putting nanostructure vertices and edges into unique concepts while realizing SiO_2_ materials. With this approach, optimization of various SiO_2_ materials for specific designs becomes reliable as distinct features of structural elements can be easier to identify. SiO_2_ materials for network design, sensor design, or other structural forms requiring sound synthesis are some areas of progressive research this focus will enable.

### Novelty of the present work

This study is practically the first that analyzes and calculates the metric and edge metric dimensions of nanosheets, nanotubes, and nanotorii of SiO_2_ in detail. With the use of graph theory, we derive the formulas to understand the structural dependence of these SiO_2_ nanostructures. Our results are not only improving scientific knowledge of these configurations but also leveraging the development of SiO2 materials with required properties. This study addresses a significant gap in the already existing studies reporting the vertices and edges in SiO_2_ nanoscopic structures. Our methodology is peculiar in that it also provides the metric dimension of these nanostructures and such knowledge may assist the progress in chemistry, materials science, and nanotechnology for the design of new materials as well as the materials with enhanced performance.

## SiO_2_ nanostructures

**Fig 1:**
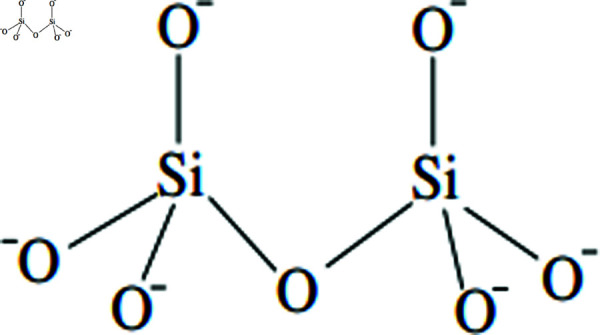
A silicon dioxide network illustrating covalent bonds between silicon and oxygen atoms.

SiO_2_, or silicon dioxide, exhibits a covalent network structure, where each silicon atom is bonded to four oxygen atoms, and each oxygen atom bonds to two silicon atoms. This connectivity creates a robust lattice structure with an overall oxygen-to-silicon atom ratio of 2:1, resulting in the molecular formula SiO_2_. Moreover, the structure shown in the Fig 1.

### SiO_2_ nanosheets

**Fig 2:**
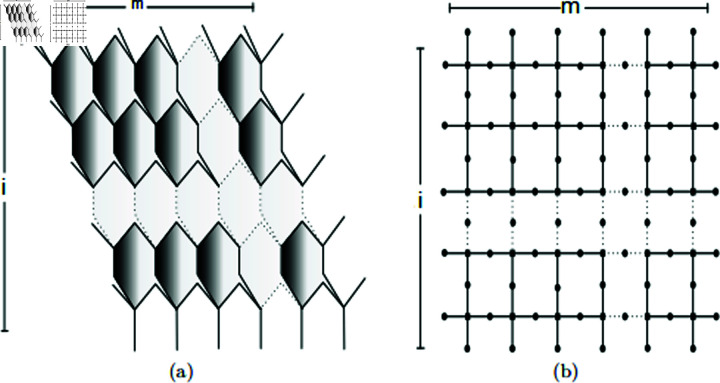
(a) Original form of SiO_2_ layer structure; (b) Brick form of SiO_2_ layer structure.

The molecules in SiO_2_ nanosheets are organized in octagonal configurations, which, when joined together, form an extensive layered structure. This arrangement is depicted in Fig 2. To facilitate the analysis of topological properties, an isomorphic representation of the SiO_2_ layer structure is also shown in Fig 2, which simplifies the computation of topological indices. In this configuration, the length and width of the nanosheet can be represented by the parameters *p* and *q* ,  denoting the number of rows and columns, respectively. Moreover, the structure shown in the Fig 2.

Prior studies have calculated various degree-based topological indices for SiO_2_ nanosheets [56–59]. In the present work, we focus on computing distance-based topological indices for different classes of SiO_2_ nanostructures, including nanosheets, which offer insights into their structural properties and potential applications [60]. For further readings, we suggest to see [61–63].

### SiO_2_ nanotubes

SiO_2_ nanotubes are tubular structures formed by curving the SiO_2_ nanosheets. These nanotubes possess a hollow core, which provides a unique internal space suitable for applications in drug delivery, nanofluidics, and adsorption-based processes. The distinctive tubular form of SiO_2_ nanotubes allows them to support functionalities that are challenging for other nanostructures. Additionally, the curvature of the structure affects certain topological indices, which can be analyzed through graph-theoretic methods to better understand their mechanical and electronic properties.

### SiO_2_ nanotori

SiO_2_ nanotori are less frequently known nanostructure of silicon dioxide which however is based on bending synthesised SiO_2_ nanotubes into a toroidal (bungalow-like) form. The structure has recently been noticed due to its applicability in the optical and photothermal correct, such as plasmonic resonance and targeted drug delivery application. The structure of SiO_2_ nanotori has a circular topology that also affects the topological properties for the intended applications in photonic and catalysis applications.

### Topological indices and metric dimension analysis

The topological indices have been previously employed in other analyses where the structural characteristics of SiO_2_ nanosheets, nanotubes, and nanotori were assessed based on the figures of symmetry/ connectivity [48]. In this work, we extend our analysis by also considering the metric dimension (MD) and edge metric dimension (EMD) of these nanostructures. The MD gives the exact number of vertices required to identify other vertices in the network besides getting the EMD to ascertain the identification of edges. The present work also seeks to improve the knowledge of the kind of SiO_2_ nanostructures by calculating these indices that will facilitate the development of materials with desired characteristics for certain applications shown in nanotechnology, material science, and chemistry [49].

** Theorem 1.** *Let* $G_{i,m}$ *denote the graph of an* *i* × *m* *two-dimensional* ${\text{SiO}}_{2}$  ( *i* , *m* )  *layer structure of Original form. The MD of an* *i* × *m* ${\text{SiO}}_{2}$  ( *i* , *m* )  *layer structure of the Original form is found to be 2.*

*Proof.* In this context, *i* and *m* represent the number of rows and columns, respectively. Consider the set of vertices:


$$\begin{array}{llll} & \{p_{\xi (1,1)},p_{\xi (1,2)},p_{\xi (1,3)},\ldots ,p_{\xi (1,m)},p_{\xi (2,1)},p_{\xi (2,2)},p_{\xi (2,3)},\; & & \;\\ & \;\ldots ,p_{\xi (2,m)},\ldots ,p_{\xi (i,1)},p_{\xi (i,2)},p_{\xi (i,3)},\ldots ,p_{\xi (i,m)}\}\; & & \; \end{array}$$

**Fig 3:**
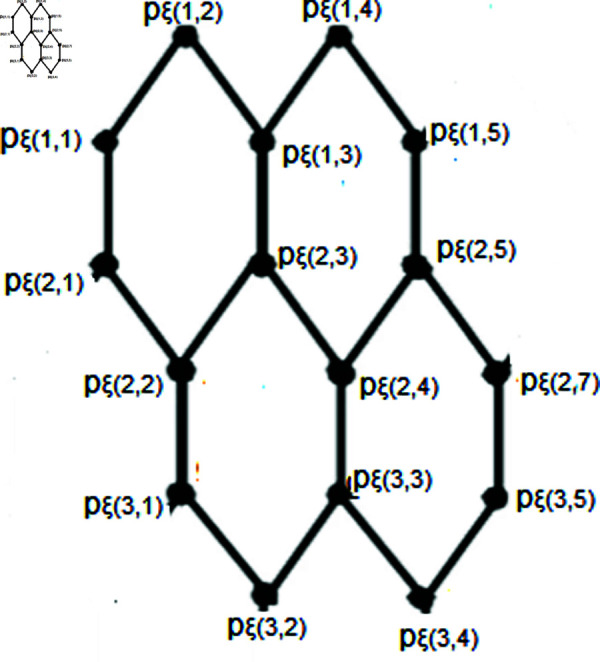
${\text{SiO}}_{2}(\text{i, m})$layer structure for *n* = 2.

Let $T=\{p_{\xi (1,1)},p_{\xi (1,2n+1)}\}$. We will demonstrate that the set *T* serves as a resolving set. Depiction shown in the Fig 3. Here let *b*=*i* + *m* . 

Firstly, we consider the case when *m* is odd.

**If *m* is odd**

**First row**


$$\begin{array}{c} \begin{array}{c} r(p_{\xi (i,m)}|T)=\{\begin{array}{cc} (b-2,2n+i-m)\; & \text{for all vertices}\\ \; \end{array} \end{array} \end{array}$$ (1)

**Second row**


$$\begin{array}{c} \begin{array}{c} r(p_{\xi (i,m)}|T)=\{\begin{array}{cc} (b-2,2n+i-m)\; & \text{for all vertices except the last one}\\ (b-2,2i-3)\; & \text{for the last vertex}\\ \; \end{array} \end{array} \end{array}$$ (2)

**Third row**


$$\begin{array}{c} \begin{array}{c} r(p_{\xi (i,m)}|T)=\{\begin{array}{cc} (b-1,2n+i-m-1)\; & \text{for all vertices except the last two}\\ (b-1,2i-3)\; & \text{for the last two vertices}\\ \; \end{array} \end{array} \end{array}$$ (3)

**Fourth row**


$$\begin{array}{c} \begin{array}{c} r(p_{\xi (i,m)}|T)=\{\begin{array}{cc} (b,2n+i-m-2)\; & \text{for all vertices except the last three}\\ (b,2i-3)\; & \text{for the last three vertices}\\ \; \end{array} \end{array} \end{array}$$ (4)

**Fifth row**


$$\begin{array}{c} \begin{array}{c} r(p_{\xi (i,m)}|T)=\{\begin{array}{cc} (b+1,2n+i-m-3)\; & \text{for all vertices except the last four}\\ (b+1,2i-3)\; & \text{for the last four vertices}\\ \; \end{array} \end{array} \end{array}$$ (5)

**Sixth row**


$$\begin{array}{c} \begin{array}{c} r(p_{\xi (i,m)}|T)=\{\begin{array}{cc} (b+2,2n+i-m-4)\; & \text{for all vertices except the last five}\\ (b+2,2i-3)\; & \text{for the last five vertices}\\ \; \end{array} \end{array} \end{array}$$ (6)

The MD of the SiO_2_ (*i* × *m*) ayer structure is shown to be 2. This is demonstrated by analyzing the resolving set $T=\{p_{\xi (1,1)},p_{\xi (1,2n+1)}\}$ and observing the distances between vertices. For odd *m*, the calculations reveal a distinctive pattern in the MDs: as we progress from the first row to subsequent rows, there is a systematic increase in one term of the distance measures, specifically *b*, while the other term, 2*n* + *i* − *m*, decreases by 1 with each additional row. This pattern reflects a gradual shift in the resolving power of the chosen vertices in the set *T*, highlighting how the MD is maintained despite variations in row configurations. The increasing series of *b* and the decreasing series of 2*n* + *i* − *m* ensure that each vertex remains distinguishable within the structure, reinforcing the conclusion that two vertices are indeed sufficient to resolve all others in this specific SiO_2_ layer structure.

Next, we consider the case when *m* is even.

**If *m* is even**

**First row**


$$\begin{array}{c} \begin{array}{c} r(p_{\xi (i,m)}|T)=\{\begin{array}{cc} (b-2,2n+i-m)\; & \text{for all vertices}\\ \; \end{array} \end{array} \end{array}$$ (7)

**Second row**


$$\begin{array}{c} \begin{array}{c} r(p_{\xi (i,m)}|T)=\{\begin{array}{cc} (b-2,2n+i-m)\; & \text{for all vertices except the last one}\\ (b-2,2i-2)\; & \text{for the last two vertices}\\ \; \end{array} \end{array} \end{array}$$ (8)

**Third row**


$$\begin{array}{c} \begin{array}{c} r(p_{\xi (i,m)}|T)=\{\begin{array}{cc} (b-1,2n+i-m-1)\; & \text{for all vertices except the last two}\\ (b-1,2i-2)\; & \text{for the last three vertices}\\ \; \end{array} \end{array} \end{array}$$ (9)

**Fourth row**


$$\begin{array}{c} \begin{array}{c} r(p_{\xi (i,m)}|T)=\{\begin{array}{cc} (b,2n+i-m-2)\; & \text{for all vertices except the last three}\\ (b,2i-2)\; & \text{for the last four vertices}\\ \; \end{array} \end{array} \end{array}$$ (10)

**Fifth row**


$$\begin{array}{c} \begin{array}{c} r(p_{\xi (i,m)}|T)=\{\begin{array}{cc} (b+1,2n+i-m-3)\; & \text{for all vertices except the last four}\\ (b+1,2i-2)\; & \text{for the last five vertices}\\ \; \end{array} \end{array} \end{array}$$ (11)

**Sixth row**


$$\begin{array}{c} \begin{array}{c} r(p_{\xi (i,m)}|T)=\{\begin{array}{cc} (b+2,2n+i-m-4)\; & \text{for all vertices except the last five}\\ (b+2,2i-2)\; & \text{for the last six vertices}\\ \; \end{array} \end{array} \end{array}$$ (12)

The observed pattern of distance measures suggests that the term *b* increases had a concomitant decrease in the value of 2*n* + *i* − *m* from the first row to subsequent rows. In particular, the term *b* is increased by 1 with each grown row, while term 2*n* + *i* − *m* is also decreased by 1 in all the vertices except for the last one at the end of the row. The fact that this pattern concerns rows of the resolving set *T* suggests that the execution of this set for many rows becomes more effective in distinguishing vertices. As the number of new rows increases with the value of *b* growing while 2*n* + *i* − *m* fades, each new row facilitates the identification of more vertices, proving the ability of the resolving set to remain useful across different configurations of the SiO_2_ layer structure. The observed series also guarantees that if a resolving set of the cardinality of two can unambiguously identify the vertices in the SiO_2_ layer structure, it can also identify the base layers and other higher dimensions of the structure efficiently and with equal determinacy. Hence we have shown that the MD is indeed 2 and T to be a resolving set for the graph of a ${\text{SiO}}_{2}$  ( *i* , *m* )  layer structure. The first row to the subsequent rows. Specifically, the term *b* increases by 1 with each row, while 2*n* + *i* − *m* decreases by 1 for all vertices except those at the end of the row. This pattern indicates that the resolving set *T* becomes increasingly effective in distinguishing vertices as the number of rows increases. Each additional row allows for more vertices to be uniquely identified due to the increasing value of *b* and the decreasing value of 2*n* + *i* − *m*, demonstrating how the resolving set maintains its efficacy across different configurations of the SiO_2_ layer structure. The observed series ensures that a resolving set of cardinality 2 is sufficient to uniquely identify all vertices in the SiO_2_ layer structure, highlighting the efficiency and consistency of the resolving set across varying dimensions of the structure.

Consequently, we have demonstrated that the MD is in fact 2 and that *T* is a resolving set for the graph of a ${\text{SiO}}_{2}$  ( *i* , *m* )  layer structure. □

** Theorem 2.** *Let* $G_{i,m}$ *denote the graph of an* *i* × *m* *two-dimensional* ${\text{SiO}}_{2}$  ( *i* , *m* )  *layer structure of bricks form. The MD of an* *i* × *m* ${\text{SiO}}_{2}$  ( *i* , *m* )  *layer structure of bricks form is 4.*

*Proof.* In this context, *i* and *m* represent the number of rows and columns, respectively. Consider the set of vertices:


$$\begin{array}{llll} & \{j_{\xi (1,1)},j_{\xi (1,2)},j_{\xi (1,3)},\ldots ,j_{\xi (1,m)},j_{\xi (2,1)},j_{\xi (2,2)},j_{\xi (2,3)},\; & & \;\\ & \;\ldots ,j_{\xi (2,m)},\ldots ,j_{\xi (i,1)},j_{\xi (i,2)},j_{\xi (i,3)},\ldots ,j_{\xi (i,m)}\}\; & & \; \end{array}$$

**Fig4:**
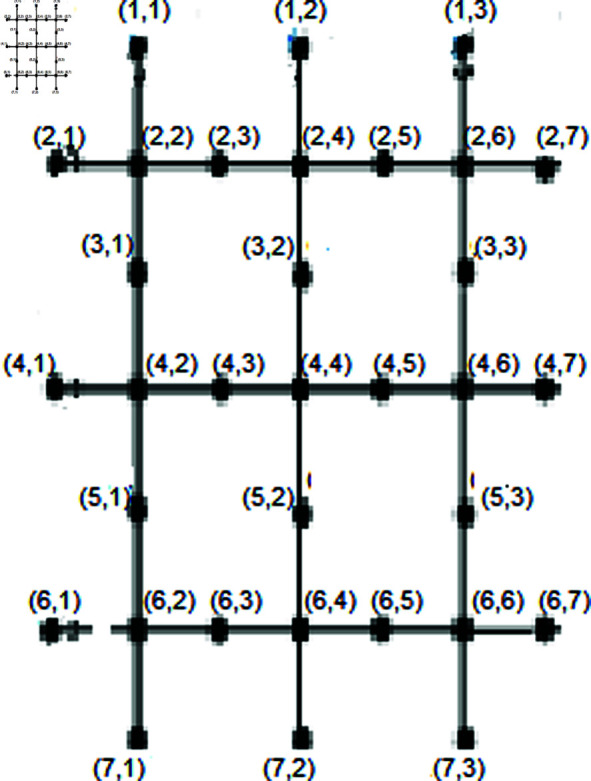
$SiO_{2}$*(i, m)* layer structure of Bricks form for *n* = 3.

Let $T=\{j_{\xi (2,1)},j_{\xi (2,2n+3)},j_{\xi (n+1,1)},j_{\xi (n+1,2n+3)}\}$. We will demonstrate that the set *T* serves as a resolving set. Depiction shown in the Fig 4.

**First row**


$$\begin{array}{c} \begin{array}{c} r(j_{\xi (i,m)}|T)=\{\begin{array}{cc} (2m,2n+(i-1)-2(m-1),2n-(i-1)\; & \\ \;+2m,5n-(i-1)-2m)\; & \text{for all }\\ \; \end{array} \end{array} \end{array}$$ (13)

**Other rows**


$$\begin{array}{c} \begin{array}{c} r(j_{\xi (i,m)}|T)=\{\begin{array}{cc} (2m+(i-3),2n+(i-1)-2(m-1),2n-(i-1)\; & \\ \;+2m,5n-(i-1)-2m)\; & \text{for all }\\ \; \end{array} \end{array} \end{array}$$ (14)

Therefore, the MD is 4. □

** Theorem 3.** *Let* $G_{i,m}$ *denote the graph of an* *i* × *m* *two-dimensional* ${\text{SiO}}_{2}$  ( *i* , *m* )  *layer structure of original form. The MD of an* *i* × *m* ${\text{SiO}}_{2}$  ( *i* , *m* )  *layer structure of bricks form is not less than 4.*

*Proof.* In this context, *i* and *m* represent the number of rows and columns, respectively. Consider the set of vertices:


$$\begin{array}{llll} & \{j_{\xi (1,1)},j_{\xi (1,2)},j_{\xi (1,3)},\ldots ,j_{\xi (1,m)},j_{\xi (2,1)},j_{\xi (2,2)},j_{\xi (2,3)},\; & & \;\\ & \;\ldots ,j_{\xi (2,m)},\ldots ,j_{\xi (i,1)},j_{\xi (i,2)},j_{\xi (i,3)},\ldots ,j_{\xi (i,m)}\}\; & & \; \end{array}$$

Assume $T=\{j_{\xi (2,1)},j_{\xi (2,2n+3)}\}$ is a resolving set. We will demonstrate that if the resolving set has cardinality 2, it leads to contradictions.

**Table:** 

Case	Vertices	Vertices
1: *n* = 2	$r(j_{\xi (1,1)}|T)=r(j_{\xi (3,1)}|T)$	$r(j_{\xi (1,3)}|T)=r(j_{\xi (3,3)}|T)$
2: *n* = 3	$r(j_{\xi (1,1)}|T)=r(j_{\xi (3,1)}|T)$	$r(j_{\xi (1,4)}|T)=r(j_{\xi (3,4)}|T)$
3: *n* = 4	$r(j_{\xi (1,1)}|T)=r(j_{\xi (3,1)}|T)$	$r(j_{\xi (1,5)}|T)=r(j_{\xi (3,5)}|T)$

Generalizing these cases, we have:


$$\begin{array}{llll} r(j_{\xi (1,1)}|T) & =r(j_{\xi (3,1)}|T),\; & & \;\\ r(j_{\xi (1,n+1)}|T) & =r(j_{\xi (3,n+1)}|T).\; & & \; \end{array}$$

These equalities indicate that a resolving set of cardinality 2 is not sufficient, as it leads to identical representations for different vertices, which is a contradiction.

Next, assume that the resolving set has cardinality 3. Let $T=\{j_{\xi (2,1)},j_{\xi (2,2n+3)},j_{\xi (n+1,1)}\}$.

**Table:** 

Case	Vertices	Vertices
1: *n* = 2	$r(j_{\xi (6,1)}|T)=r(j_{\xi (7,1)}|T)$	$r(j_{\xi (6,7)}|T)=r(j_{\xi (7,3)}|T)$
2: *n* = 3	$r(j_{\xi (8,1)}|T)=r(j_{\xi (9,1)}|T)$	$r(j_{\xi (8,9)}|T)=r(j_{\xi (9,9)}|T)$
3: *n* = 4	$r(j_{\xi (10,1)}|T)=r(j_{\xi (11,1)}|T)$	$r(j_{\xi (10,11)}|T)=r(j_{\xi (13,5)}|T)$

Generalizing these cases, we have:


$$\begin{array}{llll} r(j_{\xi (2n+2,1)}|T) & =r(j_{\xi (2n+3,1)}|T),\; & & \;\\ r(j_{\xi (2n+2,2n+3)}|T) & =r(j_{\xi (2n+3,n+1)}|T).\; & & \; \end{array}$$

These equalities indicate that a resolving set of cardinality 3 is also not sufficient, as it leads to identical representations for different vertices, which is a contradiction.

Therefore, the MD of the ${\text{SiO}}_{2}$ layer structure of bricks form is not less than 4. □

## Metric dimension of the $C_{8}$ layer structure

**Fig5:**
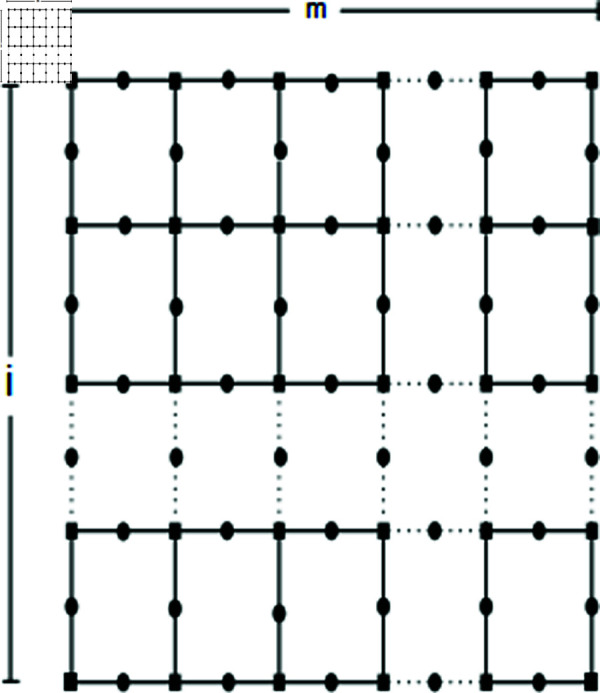
${\text{C}}_{8}$ Layer Structure.

We create an octagonal mesh as shown in Fig 5, and name this new structure the C8 layer structure of dimension  ( *i* , *m* )  by eliminating the pendent vertices and their corresponding edges from the SiO_2_ layer structure of the same dimension. This configuration has  | *V* ( *G* ) | = 3*im* + 2 ( *i* + *m* ) + 1 as many vertices and  | *E* ( *G* ) | = 4*im* + 2 ( *i* + *m* )  as many edges. After that, the C8 layer structure’s MD is examined and calculated as shown below [60,64].

** Theorem 4.** *Let* $G_{i,m}$ *denote the graph of an* *i* × *m* *two-dimensional* $C_{8}$ *layer structure of the original form. The MD of an* *i* × *m* $C_{8}$ *layer structure of the original form is found to be 2.*

*Proof.* In this context, *i* and *m* represent the number of rows and columns, respectively. Consider the set of vertices:


$$\begin{array}{llll} & \{p_{\xi (1,1)},p_{\xi (1,2)},p_{\xi (1,3)},\ldots ,p_{\xi (1,m)},p_{\xi (2,1)},p_{\xi (2,2)},p_{\xi (2,3)},\; & & \;\\ & \;\ldots ,p_{\xi (2,m)},\ldots ,p_{\xi (i,1)},p_{\xi (i,2)},p_{\xi (i,3)},\ldots ,p_{\xi (i,m)}\}\; & & \; \end{array}$$

**Fig6:**
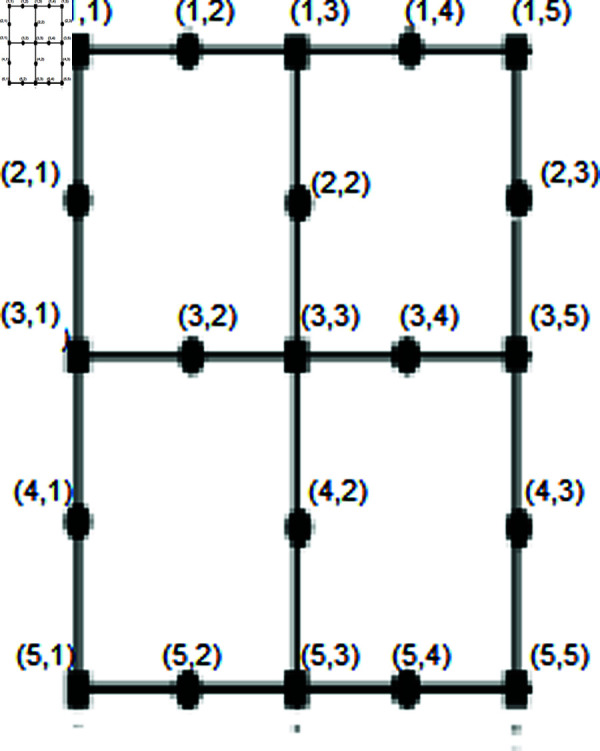
*C*_8_ Layer Structure for *n* = 2.

Let $T=\{p_{\xi (1,1)},p_{\xi (1,2n+1)}\}$. We will demonstrate that the set *T* serves as a resolving set. Depiction shown in the Figs 5 and 6. Here let *b*=*i* + *m* . 

First, we consider the case when *i* is odd:


$$\begin{array}{c} \begin{array}{c} r(p_{\xi (i,m)}|T)=\{\begin{array}{cc} (b-2,2n+i-m)\; & \text{for all vertices} \end{array} \end{array} \end{array}$$ (15)

**If *i* is even:**

For the second row, the distance representation of vertices $p_{\xi (i,m)}$ with respect to the set *T* follows a specific pattern based on the value of *m*. This pattern can be described as:


$$\begin{array}{c} \begin{array}{c} r(p_{\xi (i,m)}|T)=\{\begin{array}{cc} (b-2,2n+i-m)\; & \text{when }m=1,\\ (b-1,2n+i-m-1)\; & \text{when }m=2,\\ (b,2n+i-m-2)\; & \text{when }m=3,\\ (b+1,2n+i-m-3)\; & \text{when }m=4,\\ (b+2,2n+i-m-4)\; & \text{when }m=5,\\ (b+3,2n+i-m-5)\; & \text{when }m=6. \end{array} \end{array} \end{array}$$ (16)

In general, for any *m*, the series follows the pattern where the first component of the distance representation increments by 1 for each increase in *m*, starting from *b* − 2. Simultaneously, the second component decrements by 1 for each increase in *m*, starting from 2*n* + *i* − *m*.

Thus, we have shown that *T* is a resolving set for the graph of an $C_{8}$ )*i* × *m* )layer structure and the MD is indeed 2. □

## Metric dimension of SiO_2_ nanotube

**Fig7:**
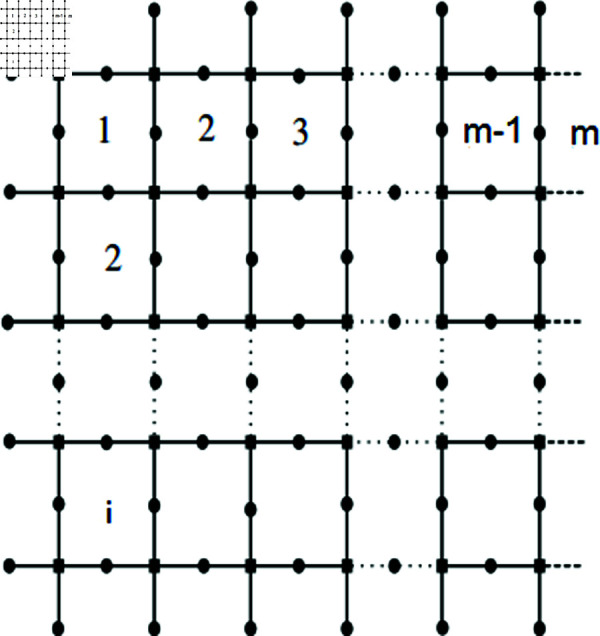
SiO_2_ nanotube (*i* ,  *m*).

Silica nanotubes have a hollow inner space that can be utilized for various functional purposes. Additionally, the mesoporous silica surface is hydrophilic, biocompatible, and suitable for functionalization, making it applicable in bioseparation, biocatalysis, biosensing, drug/gene delivery systems, adsorption, selective sequestration, drug delivery, and controlled release. Depiction shown in the Fig 7.

This section finds the MD of a SiO_2_ nanotube structure based on the known configurations of SiO_2_ sheets. To put it in graph-theoretic terms, we create a SiO_2_ nanotube of dimension  ( *p* , *q* )  by combining all pendant vertices along the left and right sides of a SiO_2_ layer structure of dimension  ( *p* , *q* − 1 ) .  This structure is shaped like a tubular structure, as shown in Fig. 7, and has  | *V* ( *G* ) | = *q* ( 3*p* + 4 )  and  | *E* ( *G* ) | = 4*q* ( *p* + 1 )  [60].

** Theorem 5.** *Let* $G_{i,m}$ *denote the graph of an* *i* × *m* *two-dimensional* SiO_2_ *nanotube. The MD of an* *i* × *m* SiO_2_ *nanotube is found to be 2.*

*Proof.* In this context, *i* and *m* represent the number of rows and columns, respectively. Consider the set of vertices:


$$\begin{array}{llll} & \{p_{\xi (1,1)},p_{\xi (1,2)},p_{\xi (1,3)},\ldots ,p_{\xi (1,m)},p_{\xi (2,1)},p_{\xi (2,2)},p_{\xi (2,3)},\; & & \;\\ & \;\ldots ,p_{\xi (2,m)},\ldots ,p_{\xi (i,1)},p_{\xi (i,2)},p_{\xi (i,3)},\ldots ,p_{\xi (i,m)}\}\; & & \; \end{array}$$

Let $T=\{p_{\xi (1,1)},p_{\xi (1,2n+2)}\}$. We will demonstrate that the set *T* serves as a resolving set. Here let *b*=*i* + *m* .  First, we consider the case when *i* is odd:


$$\begin{array}{c} \begin{array}{c} r(p_{\xi (i,m)}|T)=\{\begin{array}{cc} (b-2,2n+i-2m)\; & \text{for }m=1\\ (b-1,2n+i-2m)\; & \text{for }m=2\\ (b,2n+i-2m)\; & \text{for }m=3\\ (b+1,2n+i-2m)\; & \text{for }m=4\\ (b+2,2n+i-2m)\; & \text{for }m=5\\ \; \end{array} \end{array} \end{array}$$ (17)

**Fig8:**
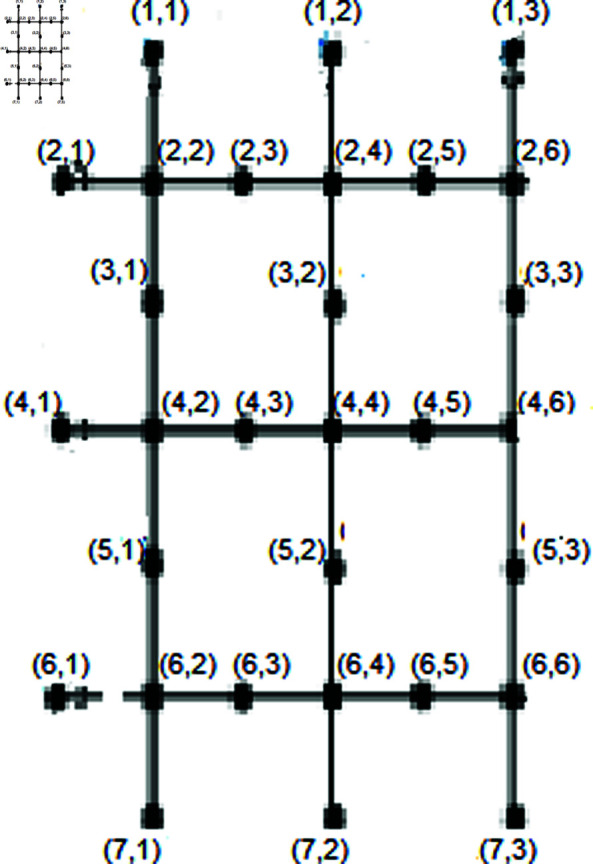
SiO_2_ nanotube (*i* ,  *m*) for *n* = 2.

The provided series represents a case where *i* is odd. It describes the function $r(p_{\xi (i,m)}|T)$, which produces different coordinate pairs depending on the value of *m*. Each value of *m* from 1 to 5 modifies the first coordinate *b* by an increment or decrement of 1 or 2. Simultaneously, the second coordinate is adjusted by subtracting 2*m* from 2*n* + *i*. This pattern indicates a systematic change in the coordinates based on the given value of *m*, illustrating a structured approach to defining the relationship between *i* and *m* in the context of the function *r*. The series can be extended by continuing this pattern for higher values of *m*. Depiction shown in the Fig 8.

**If *i* is even:**

For the first row,


$$\begin{array}{c} \begin{array}{c} r(p_{\xi (i,m)}|T)=\{\begin{array}{cc} (m-1,2n+i-m)\; & \text{for }m=1\\ (b-3,2n+i-m)\; & \text{for all} \end{array} \end{array} \end{array}$$ (18)

For other rows,


$$\begin{array}{c} \begin{array}{c} r(p_{\xi (i,m)}|T)=\{\begin{array}{cc} (b-1,2n+i-m)\; & \text{for }m=1\\ (b-3,2n+i-m)\; & \text{for all} \end{array} \end{array} \end{array}$$ (19)

Therefore, the MD of the SiO_2_ nanotube is indeed 2. □

## Metric dimension of SiO_2_ nanotori

**Fig9:**
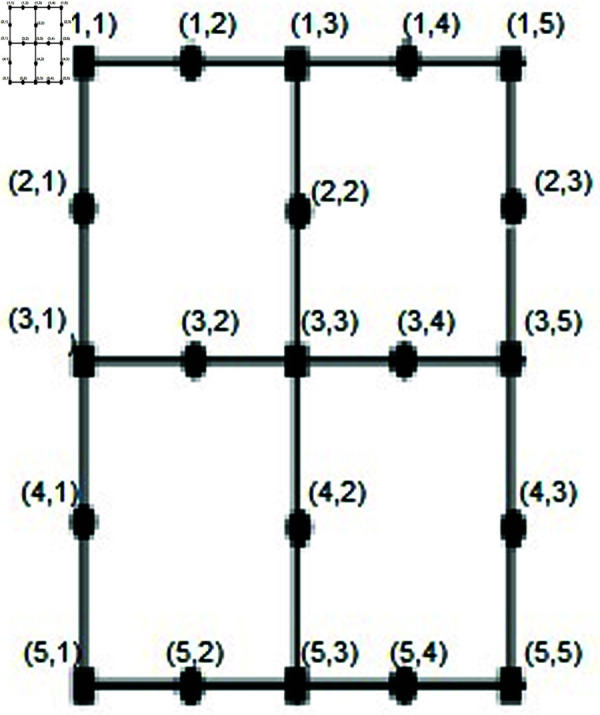
SiO_2_ nanotori (*i* ,  *m*).

Although the precise chemical structures of nanotori are currently unknown, these structures have been patented. Additionally, colloidal plasmonic gold nanotori and nanorings have been observed in pulsed laser photophysical studies. These observations are driven by the potential applications of such nanostructures, particularly inefficient photothermal drug delivery systems. When a SiO_2_ nanotube with dimensions  ( *i* − 1 , *m* )  is bent into a ring, it forms a doughnut-shaped structure that we refer to as SiO_2_ nanotori with dimensions  ( *i* , *m* ) ,  as illustrated in Figure. For this structure, the vertex count  | *V* ( *G* ) |  is 3*im* ,  and the edge count  | *E* ( *G* ) |  is 4*im* .  We now proceed to calculate the topological indices of SiO_2_ nanotori [60]. Depiction shown in the Fig 9.

** Theorem 6.** *Let* $G_{i,m}$ *denote the graph of an* *i* × *m* *two-dimensional* SiO_2_ *nanotorus. The MD of an* *i* × *m* SiO_2_ *nanotorus is found to be 3.*

*Proof.* In this context, *i* and *m* represent the number of rows and columns, respectively. Consider the set of vertices:


$$\begin{array}{llll} & \{p_{\xi (1,1)},p_{\xi (1,2)},p_{\xi (1,3)},\ldots ,p_{\xi (1,m)},p_{\xi (2,1)},p_{\xi (2,2)},p_{\xi (2,3)},\; & & \;\\ & \;\ldots ,p_{\xi (2,m)},\ldots ,p_{\xi (i,1)},p_{\xi (i,2)},p_{\xi (i,3)},\ldots ,p_{\xi (i,m)}\}\; & & \; \end{array}$$

Let $T=\{p_{\xi (1,1)},p_{\xi (1,2m+2)},p_{\xi (2m+2,1)}\}$. We will demonstrate that the set *T* serves as a resolving set. *b*=*i* + *m* First, we consider the case when *i* is odd:

For the first row,


$$\begin{array}{c} \begin{array}{c} r(p_{\xi (i,m)}|T)=\{\begin{array}{cc} (2m,3m-i-2m,3m-i-3+2m)\; & \text{for all}\\ \; \end{array} \end{array} \end{array}$$ (20)

**Fig10:**
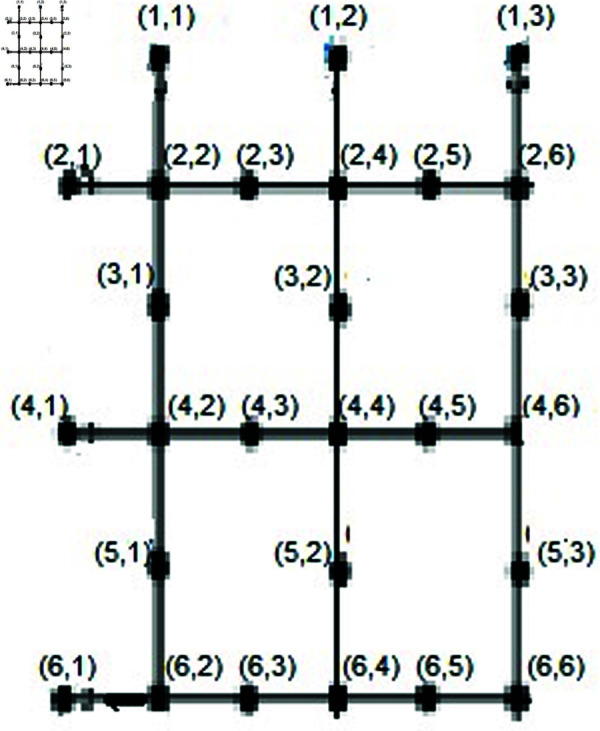
SiO_2_ nanotori (*i* ,  *m*) for *m* = 2.

For other rows, depiction shown in the Fig 10.


$$\begin{array}{c} \begin{array}{c} r(p_{\xi (i,m)}|T)=\{\begin{array}{cc} (b-2,2m+i-2m,3m-i-3+2m)\; & \text{for all}\\ \; \end{array} \end{array} \end{array}$$ (21)

**If *i* is even:**

For the first row,


$$\begin{array}{c} \begin{array}{c} r(p_{\xi (i,m)}|T)=\{\begin{array}{cc} (b-3,2m+2i-2m,2m+2i-2m)\; & \text{for }m=1\\ (b-3,2m+i-m,2m+m-1)\; & \text{for all}\\ \; \end{array} \end{array} \end{array}$$ (22)

For other rows,


$$\begin{array}{c} \begin{array}{c} r(p_{\xi (i,m)}|T)=\{\begin{array}{cc} (b-1,2m+i-m,2m+m-1)\; & \text{for }m=1\\ (b-3,2m+i-m,2m+m-1)\; & \text{for all}\\ \; \end{array} \end{array} \end{array}$$ (23)

Therefore, the MD of the SiO_2_ nanotori is indeed 3. □

The theorem asserts that the MD of a SiO_2_ nanotorus is 3, implying that any vertex within this structure can be uniquely identified by its distances to just three other specific vertices. This result is significant as it simplifies the complexity of the structure’s topology, allowing for easier analysis and potentially more efficient computational modeling. The proof demonstrates this by systematically showing that a set of three vertices can serve as a resolving set for any vertex configuration within the nanotorus. This property is particularly useful in applications such as network design, In this case, optimizing designs and functions can result from knowing the minimum number of vertices needed for unique identification.

** Theorem 7.** *Let* $G_{i,m}$ *denote the graph of an* *i* × *m* *two-dimensional* ${\text{SiO}}_{2}$ *nanotorus. The MD of an* *i* × *m* ${\text{SiO}}_{2}$ *nanotorus is at least 3.*

*Proof.* In this context, *i* and *m* represent the number of rows and columns, respectively. Consider the set of vertices:


$$\begin{array}{llll} & \{p_{\xi (1,1)},p_{\xi (1,2)},p_{\xi (1,3)},\ldots ,p_{\xi (1,m)},p_{\xi (2,1)},p_{\xi (2,2)},p_{\xi (2,3)},\; & & \;\\ & \;\ldots ,p_{\xi (2,m)},\ldots ,p_{\xi (i,1)},p_{\xi (i,2)},p_{\xi (i,3)},\ldots ,p_{\xi (i,m)}\}\; & & \; \end{array}$$

Assume $T=\{p_{\xi (1,1)},p_{\xi (2,n+2)}\}$ is a resolving set of cardinality 2. We will show that this assumption leads to contradictions.

**Table:** 

Case	Vertex Pair	Vertex Pair
1: *n* = 2	$r(p_{\xi (1,1)}|T)=r(p_{\xi (3,1)}|T)$	$r(p_{\xi (1,3)}|T)=r(p_{\xi (3,3)}|T)$
2: *n* = 3	$r(p_{\xi (1,1)}|T)=r(p_{\xi (3,1)}|T)$	$r(p_{\xi (1,4)}|T)=r(p_{\xi (3,4)}|T)$
3: *n* = 4	$r(p_{\xi (1,1)}|T)=r(p_{\xi (3,1)}|T)$	$r(p_{\xi (1,5)}|T)=r(p_{\xi (3,5)}|T)$

Generalizing these cases, we have:


$$\begin{array}{llll} r(p_{\xi (1,1)}|T) & =r(p_{\xi (3,1)}|T),\; & & \;\\ r(p_{\xi (1,n+1)}|T) & =r(p_{\xi (3,n+1)}|T).\; & & \; \end{array}$$

These equalities indicate that a resolving set of cardinality 2 is not sufficient because different vertices have identical representations, which is a contradiction.

Therefore, the MD of the ${\text{SiO}}_{2}$ nanotori is at least 3. □

## Importance of this work

Analysis of MD and EMD in SiO_2_ nanosheets, nanotubes, and nanotorii is a breakthrough for the chemistry of nanomaterials. The exact determination of these dimensions is not only crucial for revealing the specific topological characteristics of these nanostructures but also provides important insights into the design of materials and their applications in other sciences including chemistry, materials science, and nanotechnology.

This is the reason why we found it necessary to provide clear data about the MD and EMD of SiO_2_ materials in this study to meet the goals set in this study. For instance, characterizing structural elements mathematically within these nanostructures is useful knowledge that can be used to engineer or design materials with certain pre-specified traits or properties. This is especially critical as SiO_2_ is an indispensable component in a variety of technological applications ranging from electronics to catalysis, and biosensing. Such identification would enable us to fine-tune the structural elements of these materials to specific requirements of these fields particularly in terms of the conductivity, stability, or reactivity of the SiO_2_ based materials.

In addition, the results obtained from this study can be generalized beyond considering the electronic properties of the SiO_2_ nanosheets, nanotubes, and nanotorii. The presented methodologies and theoretical frameworks derived from this research could serve as a starting platform for other research on other different types of nanostructures. In this work, we draw the relationship between structural configurations of nanomaterial and metric dimensions, which enable a systematic examination of topological characteristics of corresponding nanomaterial. This is significant for the development of material science where the control of the nanostructure properties is the focal point in engineering the futuristic material. From enhancing the functions of semiconductor devices, optimizing the performance of catalysts, and developing a better understanding of nanomedicine, the information from this research will be all-encompassing and highly influential in many different fields.

Altogether, this work augments the existing databases of knowledge of the properties of nanostructured materials and offers a preliminary model for the enhancement and design of SiO_2_ materials for distinct purposes. The facility to correlate the geometric organization of nanostructures with their metric parameters opens a new attractive prospect in nanotechnology and material science developments.

## Conclusion

This study has provided an in-depth analysis of the metric and edge metric dimensions (MD and EMD) of SiO_2_ nanosheets, nanotubes, and nanotorii, offering new insights into their structural and topological characteristics. The findings reveal that the MD of SiO_2_ nanosheets is 2 in its original form, while it increases to 4 in the brick form, highlighting how structural alterations can significantly influence the metric dimensions. Additionally, the analysis of the C8 layer structure further enhanced our understanding of the complex topological properties of SiO_2_, offering a more nuanced view of its structural behavior.

These results have significant implications for practical applications in several fields, including chemistry, materials science, and nanotechnology. For instance, understanding the impact of structural changes on the MD and EMD of SiO_2_ opens up new possibilities for tailoring its properties in nanodevices, enhancing the design of advanced materials, and optimizing the synthesis processes of SiO_2_-based products. Moreover, the methodologies used in this research pave the way for similar studies on other nanostructures, offering a standardized approach to the characterization of material properties at the nanoscale.

Ultimately, the insights gained from this research contribute not only to theoretical advancements in material science but also to the practical development of SiO_2_ materials with optimized properties. By establishing a strong foundation for the future study of nanostructures, this work represents a key step in the progression of nanotechnology, providing valuable tools and frameworks for a wide range of applications in both fundamental and applied science.

## References

[1] Slater PJ. Leaves of trees. Congr. Numer. 1975;14:549–59.

[2] Harary F, Melter RA. On the metric dimension of a graph. Ars Comb. 1976;2:191–5.

[3] Hernando C, Mora M, Slater PJ, Wood DR. Fault-tolerant metric dimension of graphs. In: Proceedings of the International Conference on Convexity in Discrete Structures, Ramanujan Mathematical Society Lecture Notes; 2008. p. 81–5.

[4] Mashkaria S, Ódor G, Thiran P. On the robustness of the metric dimension of grid graphs to adding a single edge. Discrete Appl Math. 2022;316:1–27. doi: 10.1016/j.dam.2022.02.014

[5] Liu X, Ahsan M, Zahid Z, Ren S. Fault-tolerant edge metric dimension of certain families of graphs. AIMS Mathematics. 2021;6(2):1140–52. doi: 10.3934/math.2021069

[6] Walter JL, Essmann L, König SU, König P. Finding landmarks - An investigation of viewing behavior during spatial navigation in VR using a graph-theoretical analysis approach. PLoS Comput Biol 2022;18(6):e1009485. doi: 10.1371/journal.pcbi.1009485 35666726 PMC9203010

[7] Xu J, Dutta S, He W, Moortgat J, Shen H-W. Geometry-driven detection, tracking and visual analysis of viscous and gravitational fingers. IEEE Trans Vis Comput Graph. 2022;28(3):1514–28. doi: 10.1109/TVCG.2020.3017568 32809940

[8] Aversano G, Parrilla H, Bandstra M, Folsom M, Hellfeld D, Vavrek J. Data-driven event selection in pixelated cadmium zinc telluride (CZT) detectors for improved gamma-ray spectrometry. Proc. INMM Annu. Mtg. 2023.

[9] Ke Z, Liu B, Xiong W, Celikyilmaz A, Li H. Sub-network discovery and soft-masking for continual learning of mixed tasks. arXiv preprint. 2023.

[10] Thompson R. Training and evaluating graph generative models. Doctoral dissertation. 2023.

[11] Choudhary P, Bhargava L, Suhag AK. Designing of energy-efficient approximate multiplier circuit for processing unit of IoT devices. SN Comput Sci 2023;4(5):506. doi: 10.1007/s42979-023-01864-4

[12] Kampf R, Nicolaidou I. Using social impact games to overcome intractable conflicts: the case of Fact Finders and PeaceMaker. Inf Commun Soc. 2024; 1–16.

[13] Chana I, Goyal R. Computation oﬄoading techniques in edge computing: a systematic review based on energy, QoS and authentication. Concurr Comput Pract Exp. 2024:36(13);e8050.

[14] Simonraj F, George A. On the metric dimension of silicate stars. ARPN J Eng Appl Sci. 2015;5:2187–92.

[15] Siddiqui MK, Imran M. Computing the metric and partition dimension of H-Naphtalenic and VC5C7 nanotubes. J Optoelectron Adv Mater. 2015;17:790–4.

[16] Hussain Z, Munir M, Chaudhary M, Kang SM. Computing metric dimension and metric basis of 2D lattice of Alpha-Boron nanotubes. Symmetry 2018;10(8):300. doi: 10.3390/sym10080300

[17] Narducci D, Cerofolini G, Romano E. Nanotori of semiconductor material for use in diagnostics and in the anti-tumor therapy and process for the production thereof.

[18] Balaban AT. Applications of graph theory in chemistry. J Chem Inf Comput Sci. 1985;25(3):334–43.

[19] Balaban AT, Motoc I, Bonchev D, Mekenyan O. Topological indices for structure-activity correlations. Top Curr Chem. 1983;114:21–55.

[20] Balasubramanian K. A method for nuclear spin statistics in molecular spectroscopy. J Chem Phys. 1981;74(12):6824–9.

[21] Balasubramanian K. Operator and algebraic methods for NMR spectroscopy. I. Generation of NMR spin species. J Chem Phys. 1983;78(11):6358–68.

[22] Balasubramanian K. Applications of combinatorics and graph theory to spectroscopy and quantum chemistry. Chem Rev. 1985;85(6):599–618.

[23] Balasubramanian K. Characteristic polynomials of organic polymers and periodic structure. J Comput Chem. 1985;6(6):656–61.

[24] Balasubramanian K. Nuclear-spin statistics of C60, C60H60 and C60D60. Chem Phys Lett. 1991;183(3-4):292–6.

[25] Balasubramanian K. Exhaustive generation and analytical expressions of matching polynomials of fullerenes C20–C50. J Chem Inf Comput Sci. 1994;34(2):421–7.

[26] Balasubramanian K. Matching polynomials of fullerene clusters. Chem Phys Lett. 1994;201(1-4):306–14.

[27] Balasubramanian K, Khokhani K, Basak SC. Complex graph matrix representations and characterizations of proteomic maps and chemically induced changes to proteomes. J Proteome Res. 2006;5(5):1133–42. doi: 10.1021/pr050445s 16674102

[28] Balasubramanian K, Randić M. The characteristic polynomials of structures with pending bonds. Theoret Chim Acta. 1982;61(4):307–23. doi: 10.1007/bf00550410

[29] Basak SC, Grunwald GD, Gute BD, Balasubramanian K, Opitz D. Use of statistical and neural net approaches in predicting toxicity of chemicals. J Chem Inf Comput Sci. 2000;40(4):885–90. doi: 10.1021/ci9901136 10955514

[30] Basak SC, Mills D, Mumtaz MM, Balasubramanian K. Use of topological indices in predicting aryl hydrocarbon receptor binding potency of dibenzofurans: a hierarchical QSAR approach. Ind J Chem. 2003;42A(6):1385–91.

[31] Manoharan M, Balakrishnarajan M, Venuvanalingam P, Balasubramanian K. Topological resonance energy predictions of the stability of fullerene clusters. Chem Phys Lett. 1994;222(1–2):95–100.

[32] Parsons-Moss T, Schwaiger LK, Hubaud A, Hu YJ, Tuysuz H, Yang P, et al. Phosphonate-functionalized mesoporous silica. In: 241th ACS National Meeting. 2011.

[33] Ramaraj R, Balasubramanian K. Computer generation of matching polynomials of chemical graphs and lattices. J Comput Chem. 1985;6(2):122–41.

[34] Balasubramanian K. Spectra of chemical trees. Int J Quant Chem. 1982;21(3):581–90. doi: 10.1002/qua.560210306

[35] Balasubramanian K. Tree pruning and lattice statistics on Bethe lattices. J Math Chem. 1988;2(1):69–82.

[36] Balasubramanian K, Khokhani K, Basak SC. Complex graph matrix representations and characterizations of proteomic maps and chemically induced changes to proteomes. J Proteome Res. 2006;5(5):1133–42. doi: 10.1021/pr050445s 16674102

[37] Chen X, Klingeler R, Kath M, El Gendy AA, Cendrowski K, Kalenczuk RJ, et al. Magnetic silica nanotubes: synthesis, drug release, and feasibility for magnetic hyperthermia. ACS Appl Mater Interfaces. 2012;4(4):2303–9. doi: 10.1021/am300469r 22486255

[38] Horcajada P, Rámila A, Pérez-Pariente J, Vallet-Regí M. Influence of pore size of MCM-41 matrices on drug delivery rate. Microporous Mesoporous Mater. 2004;68(1–3):105–9. doi: 10.1016/j.micromeso.2003.12.012

[39] Munoz B, Rmila A, Prez Pariente J, Diaz I, Vallet-Regi M. MCM-41 organic modification as drug delivery rate regulator. Chem Mater. 2003;15(2):500–3.

[40] Vallet-Regi M, Rmila A, del Real RP, Prez Pariente J. A new property of MCM-41: drug delivery system. Chem Mater. 2001;13(2):308–11.

[41] Zhu Y, Shi J, Shen W, Dong X, Feng J, Ruan M, et al. Stimuli-responsive controlled drug release from a hollow mesoporous silica sphere/polyelectrolyte multilayer core-shell structure. Angew Chem Int Ed Engl. 2005;44(32):5083–7. doi: 10.1002/anie.200501500 16015668

[42] Azeem M, Nadeem MF. Metric-based resolvability of polycyclic aromatic hydrocarbons. Eur Phys J Plus. 2021;136(4):. doi: 10.1140/epjp/s13360-021-01399-8

[43] Nadeem MF, Azeem M, Khalil A. The locating number of hexagonal Mobius ladder network. J Appl Math Comput. 2021;66:149–65.

[44] Abbas W, Chudhary F, Farooq U, Azeem M, Shang Y. Investigating metric dimension and edge metric dimension of hexagonal boron nitride and carbon nanotubes. Eur J Pure Appl Math. 2024;17(3):2055–72. doi: 10.29020/nybg.ejpam.v17i3.5295

[45] Azeem M, Jamil MK, Javed A, Ahmad A. Verification of some topological indices of Y-junction based nanostructures by M-polynomials. J Math. 2022:2022(1);8238651.

[46] Deng B, Nadeem MF, Azeem M. On the edge metric dimension of different families of Mbius networks. Math Probl Eng. 2021:2021(1);6623208.

[47] Zuo X, Nadeem MF, Siddiqui MK, Azeem M. Edge weight based entropy of different topologies of carbon nanotubes. IEEE Access. 2021;9:102019–29. doi: 10.1109/access.2021.3097905

[48] Ahmad A-NA-H, Ahmad A, Azeem M. Computation of edge- and vertex-degree-based topological indices for tetrahedral sheets of clay minerals. Main Group Metal Chem. 2022;45(1):26–34. doi: 10.1515/mgmc-2022-0007

[49] Shanmukha MC, Lee S, Usha A, Shilpa KC, Azeem M. Degree-based entropy descriptors of graphenylene using topological indices. Comput Model Eng Sci. 2023;2023:1–25.

[50] Wang Z, Ma B-L, Gao J, Sun J. Effects of different management systems on root distribution of maize. Can J Plant Sci. 2015;95(1):21–8. doi: 10.4141/cjps-2014-026

[51] Lampurlans J, Cantero Martnez C. Soil bulk density and penetration resistance under different tillage and crop management systems and their relationship with barley root growth. Agron J. 2003;95(3):526–36.

[52] Pham HM, Yamaguchi Y, Bui TQ. A case study on the relation between city planning and urban growth using remote sensing and spatial metrics. Landsc Urban Plan. 2011;100(3):223–30. doi: 10.1016/j.landurbplan.2010.12.009

[53] Mohd I, Ahmad F, Norazriyati Wan Abd Aziz W. Exploiting town planning factors in land development. J Facilities Manag. 2009;7(4):307–18. doi: 10.1108/14725960910990053

[54] Brown A, Johnston S, Kelly K. Using service-oriented architecture and component-based development to build web service applications. Rational Softw Corp. 2002;6:1–16.

[55] Stecher P. Building business and application systems with the Retail Application Architecture. IBM Syst J. 1993;32(2):278–306. doi: 10.1147/sj.322.0278

[56] Bharali A, Bora R. Computation of some degree based topological indices of silicates (SiO2) layer. APAM. 2018;16(2):287–93. doi: 10.22457/apam.v16n2a4

[57] Farrukh F, Hafi S, Farooq R, Farahani MR. Calculating some topological indices of SiO2 layer structure. J Inform Math Sci. 2016;8(3):181–7.

[58] Farrukh F, Farooq R, Farahani MR. On the atom-bond connectivity and geometric arithmetic indices of SiO2 layer structure. Mor J Chem. 2017;5(2):384–90.

[59] Gao W, Wang W, Dimitrov D, Wang Y. Nano properties analysis via fourth multiplicative ABC indicator calculating. Arab J Chem. 2018;11(6):793–801. doi: 10.1016/j.arabjc.2017.12.024

[60] Arockiaraj M, et al. Distance-based topological indices of nanosheets, nanotubes and nanotori of SiO2. J Math Chem. 2019;57:343–69.

[61] Chen W, Li Y, Liu C, Kang Y, Qin D, Chen S, et al. In situ engineering of tumor-associated macrophages via a Nanodrug-Delivering-Drug (*β*-Elemene@Stanene) strategy for enhanced cancer chemo-immunotherapy. Angew Chem Int Ed Engl. 2023;62(41):e202308413. doi: 10.1002/anie.202308413 37380606

[62] Hao X, Jiang B, Wu J, Xiang D, Xiong Z, Li C, et al. Nanomaterials for bone metastasis. J Control Release. 2024;373:640–51. doi: 10.1016/j.jconrel.2024.07.067 39084467

[63] Long X, Chong K, Su Y, Du L, Zhang G. Connecting the macroscopic and mesoscopic properties of sintered silver nanoparticles by crystal plasticity finite element method. Eng Fract Mech. 2023;281:109137. doi: 10.1016/j.engfracmech.2023.109137

[64] Ahmad A, Koam A, Azeem M. Reverse-degree-based topological indices of fullerene cage networks. Mol Phys. 2023:121(14);e2212533.

